# Trajectory Planning of Robot Manipulator Based on RBF Neural Network

**DOI:** 10.3390/e23091207

**Published:** 2021-09-13

**Authors:** Qisong Song, Shaobo Li, Qiang Bai, Jing Yang, Ansi Zhang, Xingxing Zhang, Longxuan Zhe

**Affiliations:** 1 College of Mechanical Engineering, Guizhou University, Guiyang 550025, China; gs.qssong18@gzu.edu.cn (Q.S.); cme.qbai18@gzu.edu.cn (Q.B.); jyang23@gzu.edu.cn (J.Y.); zhangas@gzu.edu.cn (A.Z.); longxuanzhe18@163.com (L.Z.); 2 State Key Laboratory of Public Big Data, Guizhou University, Guiyang 550025, China; xingxingzhang_star@163.com

**Keywords:** robot manipulator, trajectory planning, trajectory tracking, RBF neural network, adaptive robust controller, modeling

## Abstract

Robot manipulator trajectory planning is one of the core robot technologies, and the design of controllers can improve the trajectory accuracy of manipulators. However, most of the controllers designed at this stage have not been able to effectively solve the nonlinearity and uncertainty problems of the high degree of freedom manipulators. In order to overcome these problems and improve the trajectory performance of the high degree of freedom manipulators, a manipulator trajectory planning method based on a radial basis function (RBF) neural network is proposed in this work. Firstly, a 6-DOF robot experimental platform was designed and built. Secondly, the overall manipulator trajectory planning framework was designed, which included manipulator kinematics and dynamics and a quintic polynomial interpolation algorithm. Then, an adaptive robust controller based on an RBF neural network was designed to deal with the nonlinearity and uncertainty problems, and Lyapunov theory was used to ensure the stability of the manipulator control system and the convergence of the tracking error. Finally, to test the method, a simulation and experiment were carried out. The simulation results showed that the proposed method improved the response and tracking performance to a certain extent, reduced the adjustment time and chattering, and ensured the smooth operation of the manipulator in the course of trajectory planning. The experimental results verified the effectiveness and feasibility of the method proposed in this paper.

## 1. Introduction

With the advancements in automation and robot technology, robots have begun to be widely used in the industrial, agricultural, and medical fields, among many others. Improving the trajectory planning of robot manipulators is one of the core focuses of robot research, and has great research prospects [1]. Precise robot manipulator trajectories can improve the efficiency of a robot’s various tasks, such as workshop operations, crop picking, medical surgery and so on.

A robot manipulator is a nonlinear and uncertain system. Manipulator trajectory planning should not only consider obstacle avoidance, trajectory accuracy, smooth operation, energy consumption, among other factors, but also needs to consider the problems of external interference, communication delay, and the nonlinearity and uncertainty of robot manipulators [2,3,4,5]. In order to solve these problems, many researchers have studied the kinematics formula, dynamic model, and control technology of robot manipulators. At present, research into the kinematics formula and dynamic model of robot manipulators has been gradually growing. Research into control technology has mainly focused on the sliding mode control, robust control and adaptive control, and the nonlinear and uncertain problems can be alleviated by designing controllers [6,7].

However, at present, the design of manipulator controllers is mostly based on low degree of freedom robots, and the communication delay, instability, nonlinearity and uncertainty of the high degree of freedom manipulators have not been effectively solved. These have become difficult and contentious points in current research into manipulator trajectory planning.

This study aims to promote the further development of trajectory planning research, improve the accuracy of manipulator trajectory planning, effectively deal with the nonlinearity and uncertainty of the high degree of freedom manipulators, enable manipulators to obtain good trajectory tracking performance, and better provide corresponding technical guidance for the actual trajectories of manipulators. We carried out the research on the controller design and trajectory planning of a 6-DOF robot.

A trajectory planning method for robot manipulators based on an RBF neural network is proposed in this study, which has the following contributions:

(1) The study proposes a trajectory planning method for a 6-DOF manipulator, which improves its trajectory tracking performance and motion stability, gives it higher versatility, and can be applied to the trajectory planning of a low DOF manipulator.
(2) The study designs a new adaptive robust controller based on an RBF neural network, which uses the strong robustness of adaptive control theory and the self-learning and nonlinear characteristics of RBF neural networks to deal with the nonlinearity and uncertainty of high DOF manipulators.
(3) The study designs and builds an experimental platform for the trajectory planning of manipulators and carries out the actual trajectory planning experiments based on this experimental platform, which verifies the effectiveness and feasibility of the proposed method.

The rest of this paper is organized as follows: Section 2 describes the related work on optimal trajectory planning and robot control methods. Section 3 covers the design of the trajectory planning experimental platform and introduces the overall framework of the trajectory planning method. Section 4 designs a new adaptive controller based on an RBF neural network and uses a Lyapunov function to analyze its stability and convergence. Section 5 presents the simulation and experimental results and analyzes and discusses the results. Section 6 concludes the paper and offers recommendations for future works.

## 2. Related Work

Robot manipulator trajectory planning takes the ideal trajectory kinematics parameters and the robot manipulator system as the input, and takes the displacement, velocity and acceleration of each joint and end effector as the output. The intermediate point pose is usually solved by linear interpolation [8], polynomial interpolation [9], and other interpolation algorithms.

According to differences in the planning space, trajectory planning can be divided into Cartesian space trajectory planning and joint space trajectory planning. They must both meet the kinematic and dynamic constraints of the robot manipulator, and the trajectory must be continuous, smooth, and impact-free within the performance requirements of the robot manipulator’s components; that is, the speed and acceleration must not have sudden changes [10]. At present, the research on optimal trajectory planning mainly focuses on time-optimal trajectory planning, energy-optimal trajectory planning, impact-optimal trajectory planning, and hybrid optimal trajectory planning [11,12,13].

Time-optimal trajectory planning has high work efficiency [14]. Yi Fang et al. [15] proposed a smooth and time-optimal S-curve trajectory planning method to improve the planning efficiency of manipulators. Kim et al. [16] used trapezoidal velocity curves to quickly approximate the ideal trajectory, so as to approach time-optimal planning dynamically. Zhang et al. [17] proposed an adaptive cuckoo search algorithm with faster convergence speed and higher accuracy to minimize the total motion time.

Energy-optimal trajectory planning is suitable for robots with limited energy storage, such as space exploration robots, underwater robots and military robots [18]. Liu et al. [19] used screw theory and Kane’s equations to establish kinematic and dynamic models to achieve energy optimization under the continuous motion of the manipulator. Bakshi et al. [20] optimized the robot path trajectory in multi-task environments, saving about 5–10% in energy consumption while ensuring the same work efficiency.

Impact-optimal trajectory planning aims to optimize the acceleration of each joint of the manipulator [21]. Ma et al. [22] proposed a new convex optimization method, which transforms non-convex jerk into linear acceleration and solves the acceleration limitation problem. Dai et al. [23] used a greedy algorithm to optimize the path of a robot with large jitters during manufacturing tasks, so as to improve its trajectory acceleration performance.

Hybrid-optimal trajectory planning optimizes the trajectory of the manipulator by considering time, energy consumption, impact, and other factors, and this method includes time-energy optimal, time-impact optimal, and time-impact-acceleration optimal trajectory planning [24]. Chen et al. [25] proposed an improved immune clonal selection algorithm to solve multi-objective trajectory planning. Yin et al. [26] proposed a trajectory planning method based on machine learning to generate time energy consumption optimal trajectories. Zhang et al. [27] proposed an improved dolphin swarm algorithm to generate better localization performance and more energy-efficient trajectories.

Although the above studies were able to optimize the trajectories of manipulators, they did not consider the design of the controller, failed to improve the trajectory accuracy, and did not feature the trajectory tracking error.

In [28], a robust controller based on an RBF neural network was designed to improve the trajectory tracking performance of a 3-DOF robot manipulator. In [29], an adaptive controller based on an RBF neural network was designed to solve the dynamic deviation problem of a 2-DOF robot manipulator. In [30], a sliding mode controller was designed to shorten the circular trajectory error of the 3-DOF robot manipulator. In [31], the researchers proposed a robust noise-free linear feedback control, which can effectively deal with the uncertainty of the manipulator system, suppress the external interference of the manipulator, and avoid control chattering. Ayeb et al. [32] designed an adaptive sliding mode controller based on an RBF neural network to improve the trajectory tracking performance of nonholonomic mobile robots and to avoid jitters. Al-Darraji et al. [33] designed an adaptive robust controller based on an RBF neural network, which takes into account high nonlinearity, high modeling errors, and the interference caused by payload and environmental conditions. It was able to combat effectively the nonlinear and uncertain problems of aerial robot arms. In [34], the adaptive control was used to update the parameters online in order to improve the asymptotic tracking performance of the uncertain nonlinear system, and the overall control process was introduced in detail; this description was drawn on here for the design of the controller.

## 3. Trajectory Planning Method

### 3.1. Problem Description

**Figure 1:**
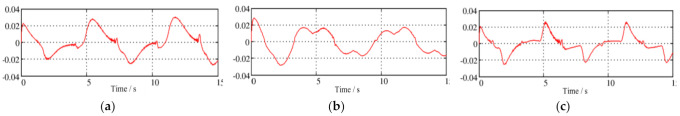
Trajectory tracking error of 3-DOF manipulator. (**a**) the tracking error of the expected trajectory and output trajectory of the first joint of the manipulator; (**b**) the tracking error of the expected trajectory and output trajectory of the second joint of the manipulator; (**c**) the tracking error of the expected trajectory and output trajectory of the third joint of the manipulator.

The design of the controllers has a significant effect on improving the trajectory tracking performance of robot manipulators. However, most of the controllers designed at this stage have been based on low degree of freedom manipulators, and the optimization of the tracking error of manipulators has also primarily been for low degree of freedom manipulators; further, and there have been error instabilities in certain trajectory optimization processes (seen in Figure 1 [28]).

Figure 1 presents the trajectory tracking error of the 3-DOF manipulator based on the RBF neural network controller. It can be seen from Figure 1 that only three manipulator joints were tracked; meanwhile, the trajectory error always exists during the trajectory operation, and does not diminish with time.

In addition, there has been little research on the design of multi-degree of freedom manipulator controllers. However, at this stage, the application scenarios of 6-DOF robots in various industries are increasing. Therefore, the trajectory optimization problem and the tracking error convergence problem of multi-degree of freedom manipulators need to be solved. It is imperative to design a controller that improves the trajectory tracking performance of 6-DOF manipulators.

### 3.2. Experiment Platform

**Figure 2:**
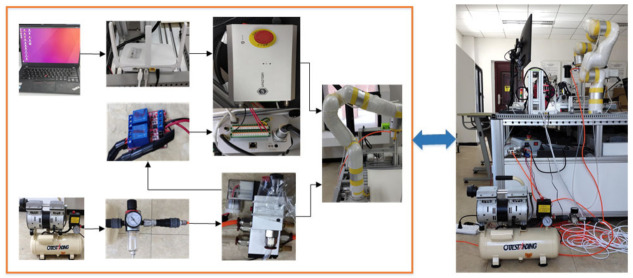
Experimental setup.

The experimental setup for manipulator trajectory planning is shown in Figure 2. The upper computer and the control cabinet were connected through a network cable, and the control cabinet and the manipulator were connected through a power supply cable and a signal cable.

**Figure 3:**
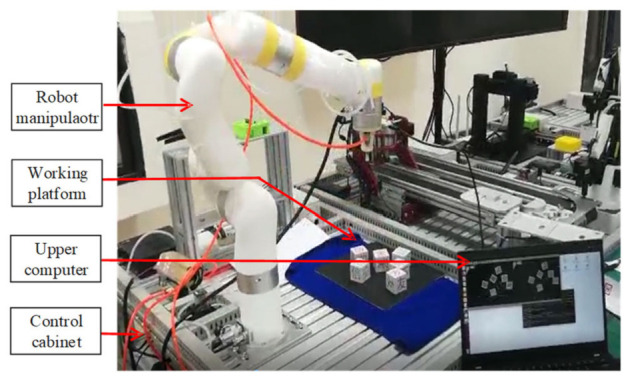
Experimental platform.

The main experimental platform for manipulator trajectory planning is shown in Figure 3, which mainly included a robot manipulator, control cabinet, upper computer and working platform.

**Figure 4:**
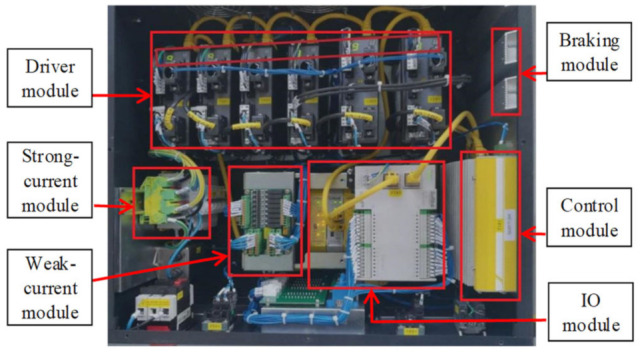
Control cabinet.

The main components of the robot control system were installed in the control cabinet. The layout of the control cabinet is shown in Figure 4, which mainly included a control module, IO module, braking module, driver module, strong-current module and weak-current module, etc.

### 3.3. Trajectory Planning Architecture

**Figure 5:**
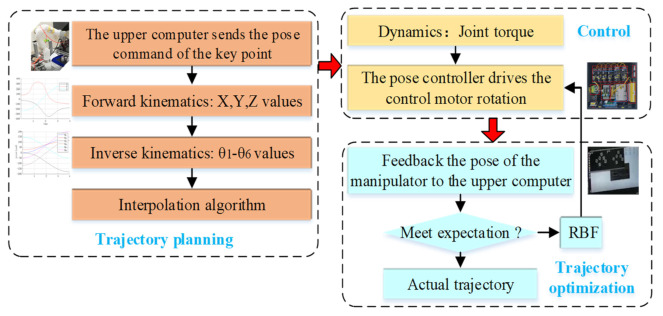
The trajectory planning structure.

The trajectory planning architecture of the robot manipulator is shown in Figure 5. This structure mainly consists of three parts.

The first stage is the trajectory planning stage. Firstly, the upper computer will send the pose commands of the key points of the robot manipulator. Secondly, the kinematics model of the manipulator is established based on the D–H method. Then, forward kinematics is used to find the x, y, and z values of the end effector, and inverse kinematics is used to find the θ_1_–θ_6_ values of joint angle. Finally, the quintic polynomial interpolation algorithm is employed to obtain the ideal trajectory.

The second stage is the control system stage. Firstly, the dynamic model of manipulator is established based on Lagrange’s theorem. Secondly, the torque of each joint under the ideal trajectory is solved by dynamics. Then, the torque information is transmitted to the pose controller. Finally, the pose controller drives the control motor rotation to realize the movement of the manipulator.

The third stage is the trajectory optimization stage. Firstly, whether the pose of the end effector reaches the expected trajectory should be assessed: if it reaches the expected trajectory, the current trajectory should be taken as the actual trajectory; otherwise, the next step should be executed. Secondly, an adaptive robust controller based on an RBF neural network is designed, and the stability and convergence of the controller are analyzed based on a Lyapunov function. Then, the designed controller is used to optimize the tracking trajectory, and the new control command is transmitted to the pose controller. Finally, the trajectory for reaching the expected goal is taken as the actual trajectory of the manipulator.

### 3.4. Modeling

#### 3.4.1. Kinematics Analysis

Position and angle analysis are the two main parts of kinematics modeling. Firstly, the structural parameters and link coordinate system of the manipulator are obtained based on the D–H method [35]. Secondly, the position of the end effector of the manipulator is obtained based on forward kinematics. Lastly, the angle of each joint of the manipulator is obtained based on inverse kinematics. Kinematics realizes the transformation of the manipulator’s coordinates in Cartesian space and joint space, which lays the foundation for the trajectory planning of the manipulator.

The forward kinematics equation is derived by a homogeneous transformation matrix, which can be expressed as Equation (1):

(1) T60=T10(θ1)T21(θ2)T32(θ3)T43(θ4)T54(θ5)T65(θ6)=nxoxaxpxnyoyaypynzozazpz0001

where p denote the position vector, a denotes the approach vector, o denotes the direction vector, and n denotes the normal vector.

The position can be expressed as Equation (2):

(2) P60=T10T21T32T43T54T65P

According to Equations (1)–(3) can then be obtained:

(3) $$\left\{\begin{array}{l} n_{x}=c_{1}\left[c_{23}(c_{4}c_{5}c_{6}-s_{4}s_{6})-s_{23}s_{5}s_{6}\right]+s_{1}(s_{4}c_{5}c_{6}+c_{4}s_{6})\\ n_{y}=s_{1}\left[c_{23}(c_{4}c_{5}c_{6}-s_{4}s_{6})-s_{23}s_{5}s_{6}\right]-c_{1}(s_{4}c_{5}c_{6}+c_{4}s_{6})\\ n_{z}=-s_{23}(c_{4}c_{5}c_{6}-s_{4}s_{6})-c_{23}s_{5}s_{6}\\ o_{x}=c_{1}\left[c_{23}(-c_{4}c_{5}c_{6}-s_{4}s_{6})+s_{23}s_{5}s_{6}\right]+s_{1}(-s_{4}c_{5}c_{6}+c_{4}s_{6})\\ o_{y}=s_{1}\left[c_{23}(-c_{4}c_{5}c_{6}-s_{4}s_{6})+s_{23}s_{5}s_{6}\right]-c_{1}(-s_{4}c_{5}c_{6}+c_{4}s_{6})\\ o_{z}=-s_{23}(-c_{4}c_{5}c_{6}-s_{4}s_{6})+c_{23}s_{5}s_{6}\\ a_{x}=-c_{1}(c_{23}c_{4}c_{5}+s_{23}c_{5})-s_{1}s_{4}s_{5}\\ a_{y}=-s_{1}(c_{23}c_{4}c_{5}+s_{23}c_{5})+c_{1}s_{4}s_{5}\\ a_{z}=s_{23}c_{4}c_{5}-c_{23}c_{5}\\ p_{x}=c_{1}(a_{1}+a_{2}c_{2}+a_{3}c_{23}-d_{4}s_{23})\\ p_{y}=s_{1}(a_{1}+a_{2}c_{2}+a_{3}c_{23}-d_{4}s_{23})\\ p_{z}=d_{1}-a_{2}s_{2}-a_{3}s_{23}-d_{4}c_{23}) \end{array}\right.$$

where the various parameters can be described as Equation (4):

(4) $$\left\{\begin{array}{l} s_{i}=\sin(\theta_{i})\\ c_{i}=\cos(\theta_{i})\\ s_{ij}=\sin(\theta_{i}+\theta_{j})=s_{i}c_{j}+c_{i}s_{j}\\ c_{ij}=\cos(\theta_{i}+\theta_{j})=c_{i}c_{j}-s_{i}s_{j} \end{array}\right.$$

The inverse kinematics equation is obtained by the algebraic method. Each joint angle can be expressed as Equation (5):

(5) $$\left\{\begin{array}{l} \theta_{1}=A\tan2\left(p_{y},p_{x}\right)-A\tan2\left(d_{3},\pm \sqrt{p_{x}^{2}+p_{y}^{2}-d_{3}^{2}}\right)\\ \theta_{2}=A\tan2[-(a_{3}+a_{2}c_{3})p_{z}+(a_{2}s_{3}-d_{4})(c_{1}p_{x}+s_{1}p_{y})]\\ \theta_{3}=A\tan2(a_{3},d_{4})-A\tan2(K,\pm \sqrt{a_{3}^{2}+d_{4}^{2}-K^{2}})\\ \theta_{4}=A\tan2(-a_{x}s_{1}+a_{y}c_{1},-a_{x}c_{1}c_{23}-a_{y}s_{1}c_{23}+a_{z}s_{23})\\ \theta_{5}=A\tan2(s_{5},c_{5})\\ \theta_{6}=A\tan2(s_{6},c_{6}) \end{array}\right.$$

#### 3.4.2. Dynamics Analysis

Dynamics analysis forms the basis of the manipulator’s controller. To date, many methods regarding the dynamics analysis of manipulators have been developed. The common methods include the Newton Euler equation, Lagrange’s equation, Kane’s equation, Appel’s equation, Routh’s equation, and so on [36]. The dynamic model of the mechanical system is derived by Lagrange’s theorem and can be described as Equation (6):

(6) $$M(q)\overset{••}{q}+V(q,\overset{•}{q})\overset{•}{q}+G(q)+F(\overset{•}{q})+d_{s}=\tau +\tau_{e}$$

where q is the joint angular displacement vector, $\overset{•}{q}$ is the joint angular velocity vector, $\overset{••}{q}$ is the joint angular acceleration vector, M(q) is the 6 × 6 order positive definite inertia matrix, $V(q,\overset{•}{q})$ is the 6 × 6 order inertia matrix, G(q) is the 6 × 1 order gravity matrix, $F(\overset{•}{q})$ is the 6 × 1 order friction matrix, $d_{s}$ is the external interference, $\tau_{e}$ is the measurable environmental torque exerted on the robot manipulators, and τ is the control input.

Suppose that the dynamic model of the robot manipulator has unknown but bounded parameters and modeling errors; then, the robot dynamics part and the measurable environmental torque described using the RBF neural network structure can be written as follows:

(7) $$M(q)\overset{••}{q}+V(q,\overset{•}{q})\overset{•}{q}+G(q)+F(\overset{•}{q})=W^{T}H\left(q,\overset{•}{q},\overset{••}{q},t\right)$$

where W are the unknown parameters for robot manipulators, and H is the RBF neural network matrix.

The measurable environmental torque described using the RBF neural network structure can be written as follows:

(8) $$\tau_{e}=W_{e}{}^{T}H_{e}\left(x_{e}\right)=W_{e}{}^{T}H_{e}\left(q,\overset{•}{q},\overset{••}{q}\right)$$

where $W_{e}$ are the unknown parameters for robot manipulators, and $H_{e}$ is the RBF neural network matrix of the environment. This model has the general characteristics of a RBF neural network, and can describe different actual environments, including the free motion condition when $W_{e}=0$.

The optimal estimation parameter for estimating $W_{e}$ is defined as follows:

(9) $${\overset{⌢}{W}}_{e}=\arg\;\min_{W_{e}\in \;\lambda_{e0}}\left[\sup_{x_{e}\in \;\lambda_{e}}|W_{e}{}^{T}H_{e}\left(x_{e}\right)-{\overset{⌢}{W}}_{e}{}^{T}H_{e}\left(x_{e}\right)\right]$$

where $\lambda_{e0}$ is the bounded set of $W_{e}$, $\lambda_{e}$ is the bounded set of $x_{e}$, and ${\overset{⌢}{W}}_{e}$ is updated online by Equation (10) to guarantee an acceptable estimation of $W_{e}$.

(10) $$\overset{•}{{\overset{⌢}{W}}_{e}}=K_{e}F_{e}{\tilde{W}}_{e}$$

where $K_{e}>0$, $F_{e}>0$, ${\tilde{W}}_{e}$ is the environmental parameters estimation error, which can be described as follows:

(11) $${\tilde{W}}_{e}=W_{e}-{\hat{W}}_{e}$$

We set $x_{e}$ and $\tau_{e}$ as the input and output of the RBF neural network respectively. Then, the optimal estimation parameters ${\overset{⌢}{W}}_{e}$ can be obtained. In this way, we can use the non-power environmental parameters ${\overset{⌢}{W}}_{e}$ in the controller to replace the traditional environmental torque $\tau_{e}$, thereby avoiding the problem of passivity.

### 3.5. Trajectory Planning Algorithm

The trajectory planning algorithm used for the robot manipulator is a quintic polynomial interpolation algorithm [37], which can be described as Equation (12):

(12) $$\left\{\begin{array}{l} \theta (t)=a_{0}+a_{1}t+a_{2}t^{2}+a_{3}t^{3}+a_{4}t^{4}+a_{5}t^{5}\\ \overset{•}{\theta }(t)=a_{1}+2a_{2}t+3a_{3}t^{2}+4a_{4}t^{3}+5a_{5}t^{4}\\ \overset{••}{\theta }(t)=2a_{2}+6a_{3}t+12a_{4}t^{2}+20a_{5}t^{3} \end{array}\right.$$

where t denotes the time, θ(t) denotes the Angular displacement, $\overset{•}{\theta }(t)$ denotes the angular velocity, and $\overset{••}{\theta }(t)$ denotes the angular acceleration.

The constraint condition of each coefficient of the quintic polynomial interpolation algorithm is described as Equation (13):

(13) $$\left\{\begin{array}{l} \theta (t_{0})=\theta_{0}=a_{0}\\ \theta (t_{f})=\theta_{f}=a_{0}+a_{1}t_{f}+a_{2}t_{f}^{2}+a_{3}t_{f}^{3}+a_{4}t_{f}^{4}+a_{5}t_{f}^{5}\\ \overset{•}{\theta }(t_{0})={\overset{•}{\theta }}_{0}=a_{1}\\ \overset{•}{\theta }(t_{f})={\overset{•}{\theta }}_{f}=a_{1}+2a_{2}t_{f}+3a_{3}t_{f}^{2}+4a_{4}t_{f}^{3}+5a_{5}t_{f}^{4}\\ \overset{••}{\theta }(t_{0})={\overset{••}{\theta }}_{0}=2a_{2}\\ \overset{••}{\theta }(t_{f})={\overset{••}{\theta }}_{f}=2a_{2}+6a_{3}t_{f}+12a_{4}t_{f}^{2}+20a_{5}t_{f}^{3} \end{array}\right.$$

When the constraint condition is satisfied, that is, when (13) is substituted into (12), Equation (14) can then be obtained:

(14) $$\left\{\begin{array}{l} a_{0}=\theta_{0}\\ a_{1}={\overset{•}{\theta }}_{0}\\ a_{2}=\frac{{\overset{••}{\theta }}_{0}}{2}\\ a_{3}=\frac{1}{2t_{{}_{f}}^{3}}[20\theta_{f}-20\theta_{0}-(8{\overset{•}{\theta }}_{f}+12{\overset{•}{\theta }}_{0})t_{f}-(3{\overset{••}{\theta }}_{0}-{\overset{••}{\theta }}_{f})t_{{}_{f}}^{2}]\\ a_{4}=\frac{1}{2t_{{}_{f}}^{4}}[30\theta_{f}-30\theta_{0}+(14{\overset{•}{\theta }}_{f}+16{\overset{•}{\theta }}_{0})t_{f}-(3{\overset{••}{\theta }}_{0}-2{\overset{••}{\theta }}_{f})t_{{}_{f}}^{2}]\\ a_{5}=\frac{1}{2t_{{}_{f}}^{5}}[12\theta_{f}-12\theta_{0}-(6{\overset{•}{\theta }}_{f}+6{\overset{•}{\theta }}_{0})t_{f}-({\overset{••}{\theta }}_{0}-{\overset{••}{\theta }}_{f})t_{{}_{f}}^{2}] \end{array}\right.$$

## 4. RBF Neural Network

### 4.1. RBF Neural Network Architecture

**Figure 6:**
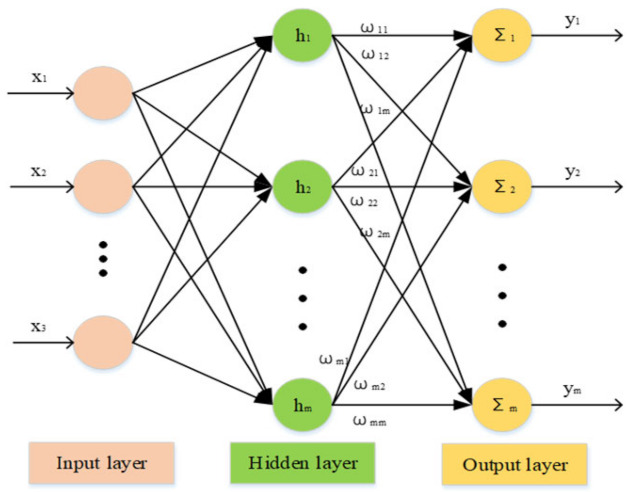
Structure of RBF neural network.

An RBF network [38] is a three-layer feedforward neural network with a radial basis function as its activation function; its structure is shown in Figure 6. It has been proven that the errors of arbitrary continuous functions can be reduced by RBF neural networks: that is, their nonlinear function approximation ability is strong. They can greatly speed up the learning rate and avoid local minima; they also have higher response speeds that are 1000 to 10,000 times faster than BP neural networks.

The three-layered RBF neural network consists of the input layer, hidden layer, and output layer. The input layer is composed of signal source nodes and transmits input excitation to the hidden layer; the hidden layer adopts gauss radial basis functions to map the low-dimensional input to the high-dimensional space and performs curve fitting; the output layer adopts a linear transformation function to perform weighted evaluation on the hidden layer signal in order to obtain the output value.

In the RBF network structure, the following notations are used [39]:

$X={\left[x_{1},x_{2},\cdots ,x_{n}\right]}^{T}$ is the input vector in the input layer, $W={\left[w_{1},w_{2},\cdots ,w_{m}\right]}^{T}$ is the weight vector, $H={\left[h_{1},h_{2},\cdots ,h_{m}\right]}^{T}$ is the radial basis vector, and $h_{j}$ is the Gaussian basis function, which can be calculated as follows:

(15) $$h_{j}=\exp\left(-\frac{{\left\|X-c_{j}\right\|}^{2}}{2\sigma_{j}^{2}}\right)\begin{array}{cc} & j=1,2,\cdots ,m \end{array}$$

where $c_{j}=[c_{1},c_{2},\cdots ,c_{m}]$ is the central vector of the *j*-th node in the network, and $\sigma_{j}$ is the mean deviation of the *j*-th node in the network. According to the structure chart, the input vector of the RBF neural network is $X={\left[x_{1},x_{2},\cdots ,x_{n}\right]}^{T}$. The output of the RBF network can be calculated as follows:

(16) $$y(t)=\sum_{i=1}^{m}w_{i}h_{i}$$

### 4.2. Adaptive Robust Controller Design Based on RBF Neural Network

In robot control, more and more researchers are designing new controllers for nonlinear problems. Because of the problems of control chattering and external interference in multi-degree of freedom manipulators, a stable controller needs to be designed.

In this study, we designed an adaptive robust controller τ based on an RBF neural network, which can achieve good tracking performance under nonlinearity and uncertainty. As indicated in the dynamic model of the system in Equation (6), the manipulator’s trajectory tracking aims to make the joint angle vector $q(t)=\;\left[q_{1}(t),q_{2}(t),\cdots ,q_{n}(t)\right]$ track the designated joint angle vector $q_{d}(t)=\;\left[q_{d1}(t),q_{d2}(t),\cdots ,q_{dn}(t)\right]$.

The trajectory tracking error and error function are defined as Equations (17) and (18), respectively.

(17) $$e(t)=q_{d}(t)-q(t)$$

(18) $$r=\overset{•}{e}+\wedge e$$

where r, ∧ is the positive diagonal matrix.

Substituting (17) and (18) into (6), Equations (19) and (20) are obtained:

(19) $$\overset{•}{q}=-r+{\overset{•}{q}}_{d}+\wedge e$$

(20) $$\begin{array}{ll} M\overset{•}{r} & =M(\overset{•}{q_{d}}-\overset{••}{q}+\wedge \overset{•}{e})=M(\overset{•}{q_{d}}+\wedge \overset{•}{e})-M\overset{••}{q}=M(\overset{•}{q_{d}}+\wedge \overset{•}{e})+V\overset{•}{q}+G+F+d_{s}-\tau -\tau_{e}\\ & =M(\overset{•}{q_{d}}+\wedge \overset{•}{e})-Vr+V(\overset{•}{q_{d}}+\wedge e)+G+F+d_{s}-\tau -\tau_{e}\\ & =-Vr-\tau -\tau_{e}+f+d_{s} \end{array}$$

where f include dynamics parameters and are usually unknown in the actual system, and they can be expressed as follows:

(21) $$f(x)=M({\overset{••}{q}}_{d}+\wedge \overset{•}{e})+V(q_{d}+\wedge e)+G+F$$

It can be seen from the above equations that an accurate mathematical model for manipulators is very necessary, but this is difficult to obtain, for manipulators are non-linear and uncertain systems. Hence, there will be a great error in the calculated result of $f\left(x\right)$. To solve this problem, an RBF neural network is used to approximate $f\left(x\right)$. Suppose the input of the RBF neural network is described as follows:

(22) $$x=\left[e^{T},{\overset{•}{e}}^{T},q_{d}^{T},{\overset{•}{q}}_{d}^{T},{\overset{••}{q}}_{d}^{T}\right]$$

The ideal output of the RBF neural network is as follows:

(23) $$h_{j}=\exp\left(-\frac{{\left\|X-c_{j}\right\|}^{2}}{2\sigma_{j}^{2}}\right)$$

(24) $$f(x)=W^{T}H(x)+\varepsilon (x)$$

where W is the weight matrix of the ideal neural network, and $\varepsilon (x)={\left[\varepsilon_{1}(x),\varepsilon_{2}(x),\cdots ,\varepsilon_{n}(x)\right]}^{T}$ is the approximation error of the ideal neural network.

Suppose the actual output of the RBF neural network is:

(25) $$\hat{f}(x)={\hat{W}}^{T}H(x)$$

Given $\tilde{W}=W-\hat{W}$,$\hat{W}$ is the weight for the actual approximation, $\tilde{W}$ is the error between the ideal weight and the actual weight, and $\hat{f}(x)$ is the actual approximation value of the neural network to f(x).

Then, the adaptive robust controller τ based on the RBF neural network can be designed as Equation (26):

(26) $$\tau ={\hat{W}}^{T}H(x)+K_{v}r-v-\tau_{e}$$

where $K_{v}>0$, and $\tau_{e}$ is the measurable environmental torque.

Meanwhile, the adaptive law for online and real-time estimation of the parameters of the radial basis function neural network can be designed as Equation (27):

(27) $$\overset{•}{\overset{⌢}{W}}=FH(x)r^{T}-KF\left\|r\right\|\overset{⌢}{W}$$

where K>0, and F>0.

According to the above expression, Equation (28) can be obtained:

(28) $$M\overset{•}{r}=-(K_{v}+V)r+\varsigma_{1}$$

where $\varsigma_{1}={\tilde{W}}^{T}H(x)+(\varepsilon +d_{s})+v$, v is the robust term used to cope with the approximation error of the RBF neural network and the system external disturbance and modeling error. v is designed as Equation (29):

(29) $$v=-(\varepsilon_{n}+b_{d})\mathrm{sgn}(r)$$

where $b_{d}$ is the upper bound of the interference error, and $\varepsilon_{n}$ is the upper bound of the approximation error: $\left\|\varepsilon \right\|\leq \varepsilon_{n}$, $d_{s}\leq b_{d}$.

In order for the robot manipulator to achieve good control performance while also ensuring stability, combined with the adaptive law and the control law with robust terms, the lower bound of $K_{v}$ must conform to the Equation (30):

(30) $$K_{v\min}\geq \frac{KW_{\max}{}^{2}}{4\left\|r\right\|}$$

Then, the position signal, velocity signal, and acceleration signal are all bounded, and after this, the robot manipulator system tends to be stable. As time increases, the tracking error gradually tends to zero: that is, $e\left(t\right)\to 0$ as t→∞.

### 4.3. Stability and Convergence Analysis

For the adaptive robust control of the robot manipulator based on the RBF neural network, the Lyapunov function [40] is defined as Equation (31):

(31) $$L=\frac{1}{2}r^{T}Mr+\frac{1}{2}tr({\tilde{W}}^{T}F^{-1}\tilde{W})$$

Then, the derivative of L can be derived as Equation (32):

(32) $$\overset{•}{L}=r^{T}M\overset{•}{r}+\frac{1}{2}r^{T}\overset{•}{M}r+tr({\tilde{W}}^{T}F^{-1}\overset{•}{\tilde{W}})$$

Substituting (28) into (31), Equation (33) can be obtained:

(33) $$\overset{•}{L}=-r^{T}K_{v}r+\frac{1}{2}r^{T}(\overset{•}{M}-2V)r+tr{\tilde{W}}^{T}(F^{-1}\overset{•}{\tilde{W}}+Hr^{T})+r^{T}(\varepsilon +d_{s}+v)$$

Substituting the adaptive law (27) into (33), we can then obtain Equation (34):

(34) $$\begin{array}{ll} \overset{•}{L} & =-r^{T}K_{v}r+r^{T}\left(\varepsilon +d_{s}+v\right)+tr{\tilde{W}}^{T}\left(-F^{-1}FHr^{T}+KF^{-1}F\left\|r\right\|\tilde{W}+Hr^{T}\right)\\ & =-r^{T}K_{v}r+r^{T}\left(\varepsilon +d_{s}+v\right)+K\left\|r\right\|tr\tilde{W}\left(W-\tilde{W}\right) \end{array}$$

where the trace of matrix $\tilde{W}\left(W-\tilde{W}\right)$ can be described as Equation (35):

(35) $$tr{\tilde{W}}^{T}\left(W-\tilde{W}\right)=\left(\tilde{W},W\right)-{\left\|\tilde{W}\right\|}^{2}\leq \left\|\tilde{W}\right\|\left\|W\right\|-{\left\|\tilde{W}\right\|}^{2}$$

Then, the relationship of $\overset{•}{L}$ can be derived as Equation (36):

(36) $$\begin{array}{ll} \overset{•}{L} & =-r^{T}K_{v}r+r^{T}\left(\varepsilon +d_{s}+v\right)+K\left\|r\right\|tr\tilde{W}\left(W-\tilde{W}\right)\\ & \leq -K_{v\min}{\left\|r\right\|}^{2}+r^{T}\left(\varepsilon +d_{s}+v\right)+K\left\|r\right\|\left\|\tilde{W}\right\|\left(W_{\max}-\left\|\tilde{W}\right\|\right)\\ & \leq -K_{v\min}{\left\|r\right\|}^{2}+r^{T}\left(\varepsilon +d_{s}\right)-\left\|r\right\|\left(\varepsilon_{n}+b_{d}\right)+K\left\|r\right\|\left\|\tilde{W}\right\|\left(W_{\max}-\left\|\tilde{W}\right\|\right)\\ & \leq -\left\|r\right\|\left[K_{v\min}\left\|r\right\|+K\left\|\tilde{W}\right\|\left(\left\|\tilde{W}\right\|-W_{\max}\right)\right]\\ & =-\left\|r\right\|\left[K\left(\left\|\tilde{W}\right\|-\frac{W_{\max}}{2}\right)-\frac{KW_{\max}{}^{2}}{4}+K_{v\min}\left\|r\right\|\right] \end{array}$$

When the lower bound of $K_{v}$ meets the Equation (30), Equation (37) can then be obtained:

(37) $$\overset{•}{L}\leq -\left\|r\right\|\left[K\left(\left\|\tilde{W}\right\|-\frac{W_{\max}}{2}\right)-\frac{KW_{\max}{}^{2}}{4}+K_{v\min}\left\|r\right\|\right]\leq 0$$

As L≥0, $\overset{•}{L}\leq 0$; therefore, L is positive and bounded. Meanwhile, $M\left(q\right)$ is also positive and bounded, which indicates that r(t), $\tilde{W}$, and $\hat{W}$ are also bounded. Moreover, when the value of the Lyapunov function is equal to 0, the value of the error function is also equal to 0: that is, r=0 when $\overset{•}{L}=0$. According to Barbalat’s lemma, the robot manipulator system is asymptotically stable. Therefore, as time increases, the tracking error, the derivative of the tracking error, and the error function all also gradually tend to zero: that is, $e\left(t\right)\to 0$, $\overset{•}{e}(t)\to 0$, and r→0 as t→∞.

## 5. Simulation and Experiment Results

### 5.1. Trajectory Tracking Simulation

#### 5.1.1. Simulation Results

**Figure 7:**
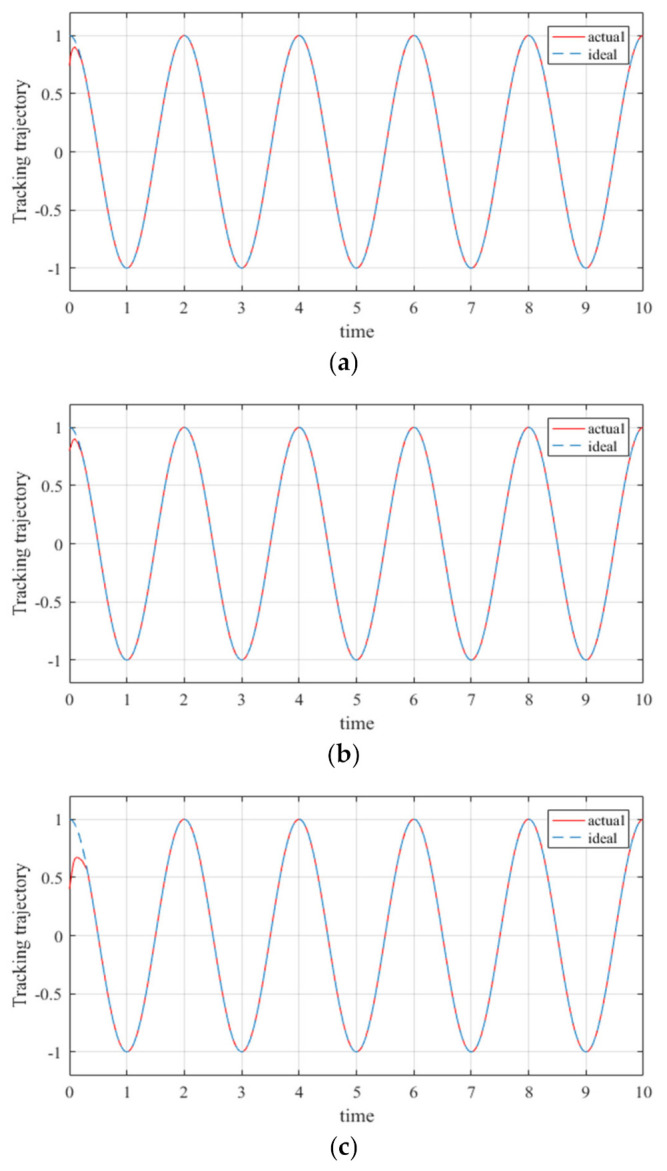

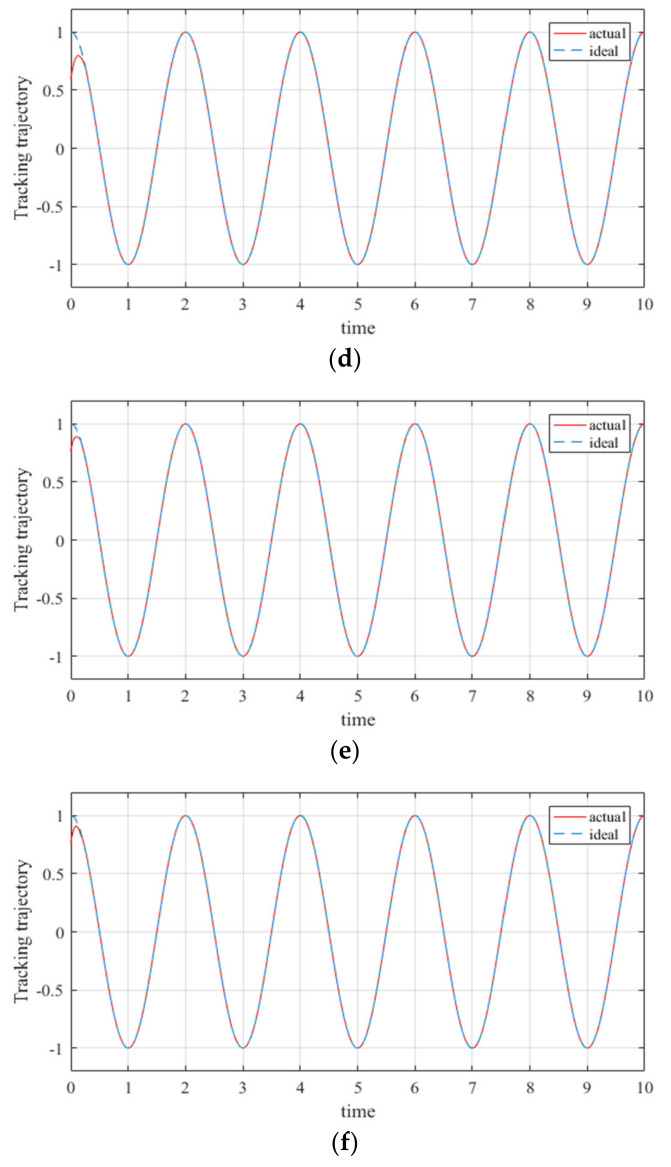
Joint angle tracking of the proposed controller: (**a**) angle tracking of joint 1; (**b**) angle tracking of joint 2; (**c**) angle tracking of joint 3; (**d**) angle tracking of joint 4; (**e**) angle tracking of joint 5; (**f**) angle tracking of joint 6.

**Figure 8:**
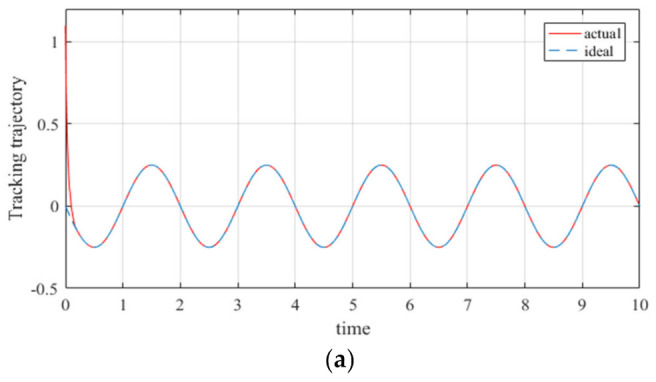

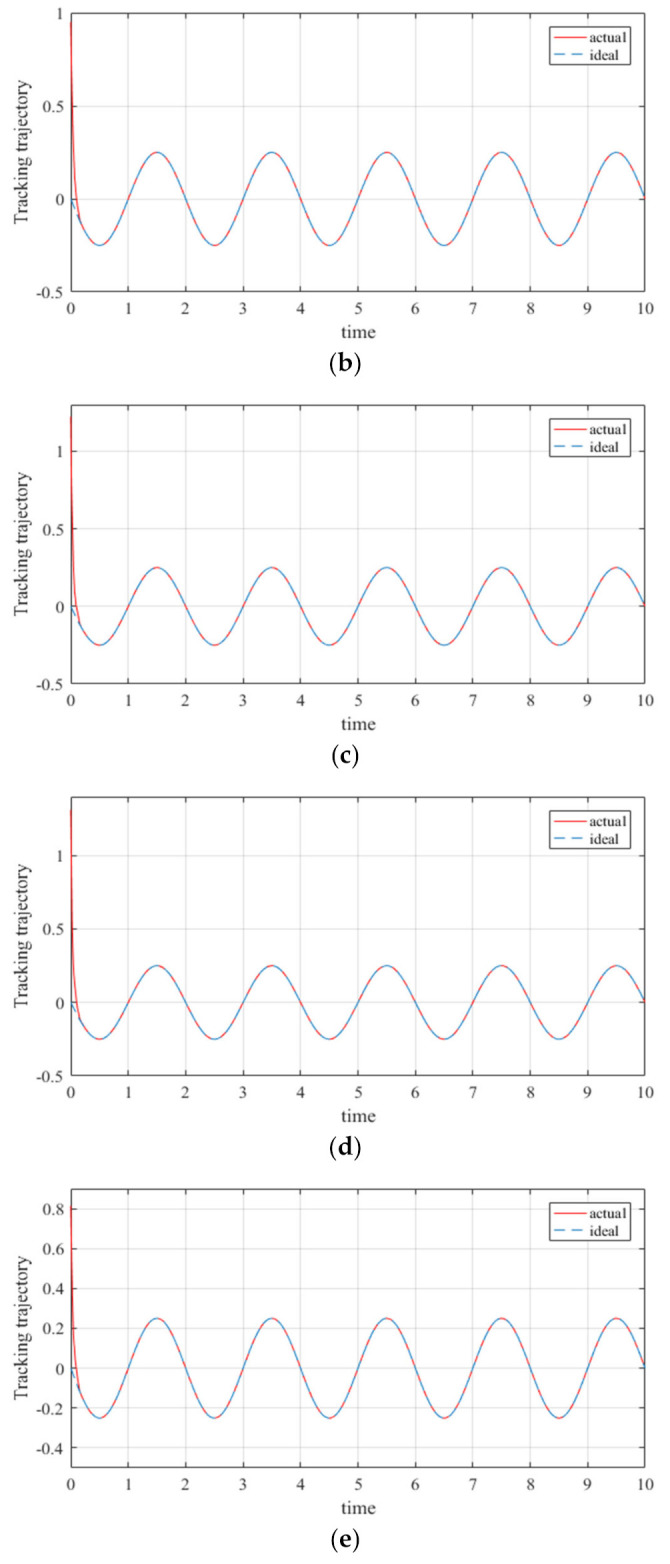

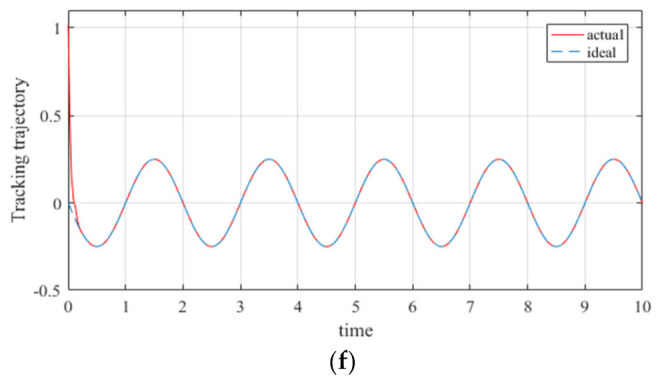
Joint position tracking of the proposed controller: (**a**) position tracking of joint 1; (**b**) position tracking of joint 2; (**c**) position tracking of joint 3; (**d**) position tracking of joint 4; (**e**) position tracking of joint 5; (**f**) position tracking of joint 6.

The trajectory tracking simulation results are shown in the figures below. Figure 7 shows the trajectory of the joint angle tracking of the adaptive robust controller based on the RBF neural network. Figure 8 shows the trajectory of the joint position tracking of the adaptive robust controller based on the RBF neural network. The red line represents the actual trajectory, and the blue dotted line represents the ideal trajectory.

#### 5.1.2. Simulation Analysis

Based on the simulation results, the following conclusions can be reached.

(1) In Figure 7 and Figure 8, the estimation f(x) with the RBF neural network is expressed by $\hat{f}(x)$. We can see that $\hat{f}(x)$ almost approximated to f(x) after 0.3 s, and the error was in an acceptable range.
(2) It can be seen from Figure 7 and Figure 8 that the adaptive robust controller based on the RBF neural network tracked the trajectory of the manipulator, and therefore the proposed controller improved the response time while reducing the adjustment time.
(3) The tracking errors in the simulation strictly converged to zero; this means that the proposed controller can guarantee the stability of manipulators in real applications. In other words, the simulation results reveal that the proposed controller is effective for multi-degree of freedom manipulators faced with uncertainties and external disturbances.

Meanwhile, the controller proposed in this study was compared with other controllers, and the comparison results are shown in Table 1.

From Table 1, it can be seen that the controller designed in this study not only considers joint angle tracking and joint position tracking in the trajectory tracking of 6-DOF manipulators, but also considers the external environment interference to ensure the credibility of their trajectories. Compared with other controllers, the trajectory tracking error of this controller is not the smallest, but the error approximation time is very short, and can be applied to a 6-DOF manipulator.

#### 5.1.3. Simulation Discussion

In the process of manipulator trajectory tracking, there was only a large error in the initial state, and the error decreased rapidly in a very short time. Then, the trajectory tracking error gradually converged to 0, and there was no sudden change in the error. This indicates that the adaptive robust controller based on the RBF neural network designed in this study achieved good results in realizing the trajectory tracking of the 6-DOF manipulator, has extremely high stability, and improves the trajectory tracking performance of the manipulator.

### 5.2. Trajectory Planning Simulation

#### 5.2.1. Simulation Results

It has been proven that the RBF neural network can fit discrete points with minimal errors, so it can therefore be applied to manipulator trajectory planning. We adopted the quintic polynomial interpolation algorithm to plan the trajectory of the robot manipulator.

**Figure 9:**
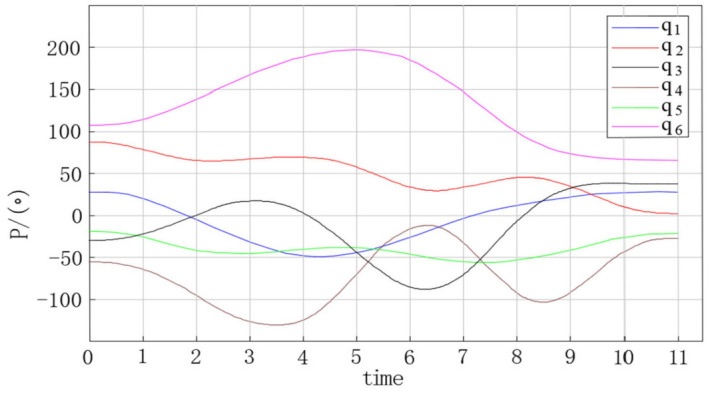
Angular displacement curve of each joint of the manipulator.

**Figure 10:**
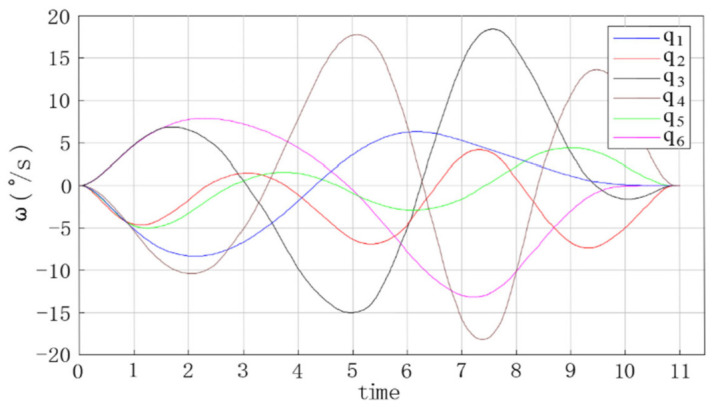
Angular velocity curve of each joint of the manipulator.

**Figure 11:**
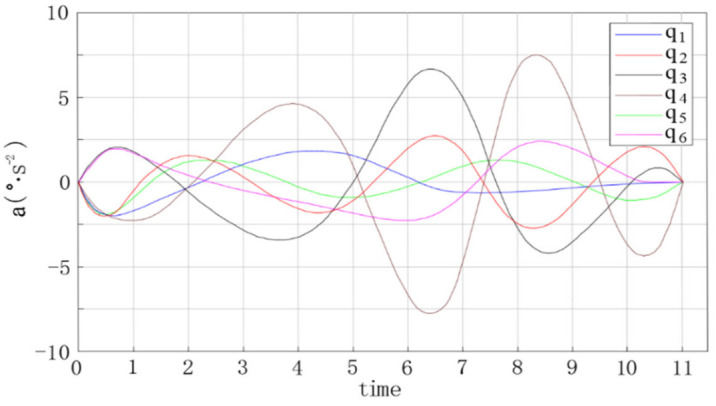
Angular acceleration curve of each joint of the manipulator.

The trajectory planning simulation results are shown in the figures below. Figure 9 shows the angular displacement trajectory of each joint of the robot manipulator. Figure 10 shows the angular velocity trajectory of each joint of the robot manipulator. Figure 11 shows the angular acceleration trajectory of each joint of the robot manipulator.

#### 5.2.2. Simulation Analysis

Based on the trajectory planning simulation results, the following conclusions can be reached.

(1) In Figure 9, Figure 10 and Figure 11, the trajectories of the joint angular displacement, angular velocity, and angular acceleration are smooth, and there are no jumps. This indicates that there were no jitter or impact problems in the trajectory planning of the multi-degree of freedom manipulator.
(2) It can be seen from Figure 10 and Figure 11 that the angular velocity and angular acceleration of each joint of the manipulator at the starting and ending position were all 0, which indicates that the manipulator can run smoothly when starting and stopping movement, as well as during the entire process of completing work tasks.
(3) It can be seen from Figure 10 and Figure 11 that the velocity and acceleration of joint 3 and joint 4 of the 6-DOF manipulator varied drastically from 3 to 9 s, indicating that the method proposed in this study can improve the trajectory planning speed and shorten the trajectory planning time.

#### 5.2.3. Simulation Discussion

During the trajectory planning of the 6-DOF robot manipulator, the positions of the six joints were constantly changing, which indicates that the method proposed in this paper can realize the full scheduling of the 6-DOF robot manipulator. The velocity and acceleration values of the initial state and the end state of the trajectory planning were all 0, which represents the smooth end of a trajectory planning experiment. The position, velocity, and acceleration curves of the trajectory planning were smooth, indicating that the method proposed in this paper can effectively improve the stability, speed, and accuracy of trajectory planning.

### 5.3. Trajectory Planning Experiment

#### 5.3.1. Experiment Results

The trajectory planning experiment data of the manipulator passing through 12 space nodes are shown in Table 2, which shows the actual variables of each joint and the coordinates of the end effector under the 12 space nodes of the manipulator.

**Figure 12:**
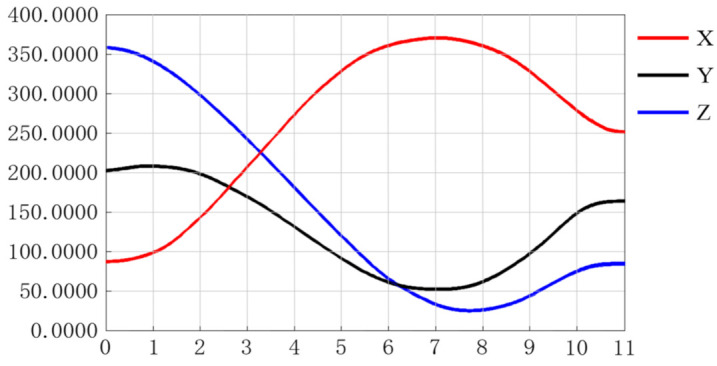
End effector displacement curve.

According to the Cartesian space coordinates in Table 2, the *x*-axis, *y*-axis, and z-axis displacement curves of the end effector of the manipulator are drawn using the quintic polynomial interpolation function, as shown in Figure 12.

**Figure 13:**
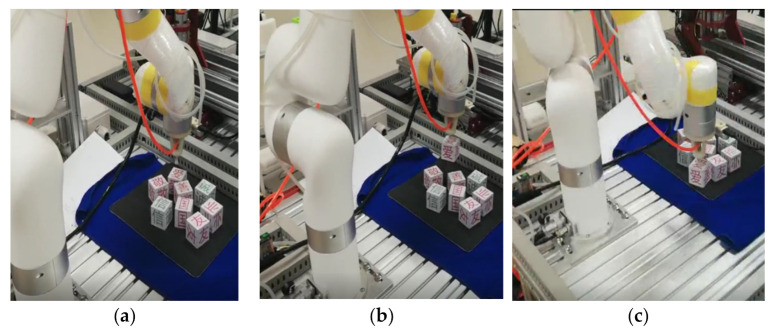

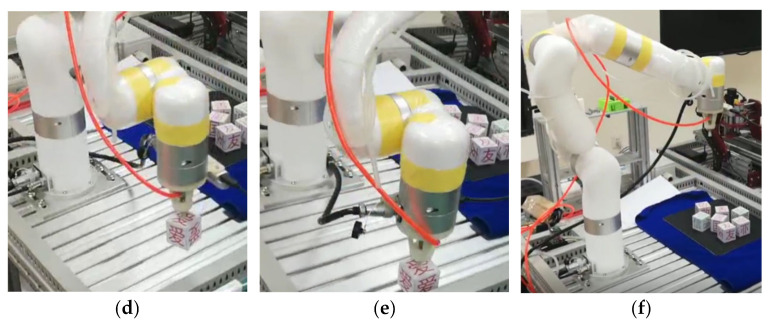
Trajectory planning experiment of the manipulator: (**a**–**f**) represent different states of the manipulator in the process of trajectory planning.

Meanwhile, the overall process of the manipulator trajectory planning experiment is shown in Figure 13. 

#### 5.3.2. Experiment Analysis

Based on the trajectory planning simulation results, the following conclusions can be reached.

(1) It can be seen from Figure 12 that the three coordinate changes of the end effector were smooth curves, which indicates the rationality of the forward and inverse kinematics model designed in this paper.
(2) Figure 13a represents the initial state of manipulator trajectory planning, Figure 13b represents the state when picking up the object, Figure 13c,d represents the state of the moving trajectory, Figure 13e represents the state when the object is placed, and Figure 13f represents the end state of the trajectory.
(3) It can be seen from Figure 13 that the manipulator ran smoothly, and the trajectory was smooth and continuous in the wood block grasping experiment, which verifies the feasibility of the method proposed in this paper.

#### 5.3.3. Experiment Discussion

The trajectory planning experiment for the 6-DOF manipulator was conducted to verify the feasibility of the method proposed in this paper. In the actual experiment, it ran smoothly without sudden changes in speed, and could grasp the target, which indicates that the method proposed in this paper not only has theoretical significance, but also has practical application value. It can make up for part of the current gap in the manipulator research field and can provide corresponding technical theoretical support for multi-degree of freedom manipulator trajectory planning.

## 6. Conclusions

In this paper, we proposed a powerful trajectory planning method for robot manipulators, which is based on an RBF neural network. The proposed method was evaluated by two simulations. The first simulation evaluated the precision of the trajectory tracking of the manipulator, and the second simulation evaluated the motion stability of the trajectory planning of the manipulator. In addition, the proposed method was verified by an experiment. The experiment not only verified the rationality of the kinematics and dynamics model, but also verified the feasibility and effectiveness of the proposed method. The simulation and experiment results proved that the proposed method can improve the trajectory tracking accuracy and motion efficiency of the manipulator. Meanwhile, the designed controller is robust, able to withstand not only external disturbances but also parameter uncertainties. This paper focuses on the trajectory planning of multi-degree of freedom manipulators and makes corresponding explorations into the development of robots.

In the future, further tests are essential for performance evaluation of the proposed control approach. We will take the motion time and energy consumption into account to obtain a comprehensive optimal trajectory. Meanwhile, we will also continue to study and optimize the control strategies of manipulators.

## Figures and Tables

**Table 1:** Controller comparison results.

Controller	Manipulator	Maximum Tracking Error	Error Approach Time	Joint Position Tracking	Joint Angle Tracking	Environmental Interference
Robust controller [28]	3 DOF	0.028 rad	Near 1 s	No	Yes	Not considered
Adaptive controller [29]	2 DOF	Near 0.4 rad	Near 6 s	No	Yes	Not considered
Sliding mode controller [30]	2 DOF	Near 0.7 rad	Near 2.5 s	Yes	No	Not considered
Adaptive sliding controller [41]	2 DOF	Near 0.43 rad	Near 0.6 s	No	Yes	Considered
Proposed controller	6 DOF	Near 0.5 rad	Near 0.2 s	Yes	Yes	Considered

**Table 2:** Manipulator motion data.

Position	Actual Joint Variables (°)						End Effector Coordinates (mm)		
	$\theta_{1}$	$\theta_{2}$	$\theta_{3}$	$\theta_{4}$	$\theta_{5}$	$\theta_{6}$	X	Y	Z
0	32.0364	84.3602	33.0139	−52.6213	−21.3309	103.4506	78.2096	202.0341	357.6408
1	21.4961	80.1347	23.8134	−61.2139	−25.6102	109.1382	99.8657	208.3627	339.5047
2	−3.6823	67.2143	0.9148	−96.0141	−45.8168	142.8134	146.3812	199.0349	298.7648
3	−34.3147	68.8139	20.6127	−124.4116	−47.2314	168.5148	207.9973	171.3106	243.3942
4	−48.3015	70.5126	1.8631	−123.1671	−44.1861	183.7152	273.6841	132.6172	179.3016
5	−46.1137	61.8217	−46.2916	−71.1029	−43.2319	192.3185	332.4615	89.3364	122.3657
6	−23.6124	42.8133	−86.1991	−11.7163	−48.3161	180.7162	362.5973	62.8249	65.8143
7	−0.8135	42.2019	−71.8634	−31.9013	−52.3172	148.6173	368.7526	52.6815	36.4265
8	16.2643	47.9103	−9.8638	−47.3129	−51.0192	109.8167	363.0214	63.8462	28.8318
9	23.6245	43.1081	42.4813	−47.6013	−44.5148	73.9216	330.8106	98.1835	48.2154
10	28.3012	13.2164	43.8156	−48.1176	−24.6137	19.2131	278.6105	149.0263	77.3167
11	29.6148	1.5018	43.8156	−16.9135	−23.1772	13.2131	251.0648	166.8019	83.3182

## Data Availability

Not applicable.
